# The Influence of Cleaning Solutions on the Retention of Overdenture Attachment Systems

**DOI:** 10.3390/biomedicines11061681

**Published:** 2023-06-09

**Authors:** Sofia Monteiro, Pedro Barreiros, Joana Mendes, Carlos Aroso, António Sérgio Silva, José Manuel Mendes

**Affiliations:** 1 Oral Rehabilitation and Prosthodontics Service, University Institute of Health Sciences (IUCS), CESPU, 4585-116 Gandra, Portugal; a22593@alunos.cespu.pt; 2 UNIPRO—Oral Pathology and Rehabilitation Research Unit, University Institute of Health Sciences (IUCS), CESPU, 4585-116 Gandra, Portugal; joana.silva.mendes@iucs.cespu.pt (J.M.); carlos.ribeiro@iucs.cespu.pt (C.A.); asergio.silva@iucs.cespu.pt (A.S.S.); jose.mendes@iucs.cespu.pt (J.M.M.)

**Keywords:** denture cleanser, attachments, overdenture, oral health, quality of life

## Abstract

Dental-implant-supported reconstructions provide comfort and improvements in prosthetic function, adaptation, and stability over conventional treatment options. The objective of this study was to evaluate the effect of different denture cleansing solutions and their influence on the deterioration and loss of retention of overdenture attachments in a 12-month clinical-use simulation. In this way, ten specimens each of different brands of retentive caps made of Teflon (OT Equator^®^ (Rhein83, Bologna, Italy), Locator^®^ (Zest Anchors, Escondido, CA, USA), Kerator^®^ (KJ Meditech, Gwangiu, Republic of Korea), and Locator R-Tx^®^ (Zest Anchors, Escondido, CA, USA)) were immersed in five different cleaning solutions (Kukident^®^ (P&G Tech, Oxford Parkway, UK), Benfix^®^ (Laboratorios URGO S.L., Guipúzcoa, Spain), Corega^®^ (Stafford Miller, Waterford, Ireland), and Protefix^®^ (Neuhofer Weiche, Parchim, Germany)), and tap water was used as the control group, in a simulation that lasted 12 months. Data were analyzed using two-way ANOVA and a Tukey HSD. Furthermore, a Levene Test and Shapiro–Wilk tests were performed to assess the validation of the ANOVA assumptions. The statistical analysis was performed using R version 4.2.2 software with the significance level set to *p* < 0.05. There were significant statistical differences between the different manufacturers regarding the retention forces of the attachment’s retentive caps (F = 322.066, *p* < 0.001). For the cleaning solution groups, different statistical results between Kukident^®^ (P&G Tech, Oxford Parkway, UK) (*p* < 0.05) and Benfix^®^ (Laboratorios URGO S.L., Guipúzcoa, Spain) (*p* < 0.05) were observed. There were no significant statistical differences between Corega^®^ (Stafford Miller, Ireland), Protefix^®^ (Neuhofer Weiche, Parchim, Germany), and tap water, even though the retention forces decreased in all of them.

## 1. Introduction

Despite continuous improvements in oral health worldwide, edentulism continues to be an irreversible and debilitating condition that is prevalent in several different countries and especially in elderly communities [1,2]. Therefore, it is crucial to implement treatment measures for the edentulous population in order to reduce the numbers of those suffering from this condition, and the development and improvement of prosthodontic techniques is mandatory [3,4,5].

The most common treatments for edentulous patients are muco-supported and dento-muco-supported prostheses [3]. However, the success of conventional complete denture therapy is directly affected by the oral anatomy, which can lead to a lack of retention and stability and affect mastication and speech. Nevertheless, the need to improve the function of the remaining teeth with fixed options, together with the increase in treatment options through implants, has led to a greater acceptance and demand for prostheses that use implants to retain and support them [3,4,5]. Dental-implant-supported reconstructions have also become a frequent treatment option for the treatment of partially and fully edentulous jaws [6,7,8]. Full-arch implant-supported fixed dental prostheses provide some advantages over conventional treatment options, such as comfort, substantial improvements in prosthetic function, adaptation, and stability [4,6,7,8,9,10,11,12]. This type of treatment requires good oral hygiene to minimize the risk of peri-implant infections, as further complications may still arise. In fact, there is strong evidence from longitudinal and cross-sectional studies that point to an increased risk of developing peri-implantitis in patients with a history of periodontitis and who have lost their teeth [13,14,15].

Peri-implant diseases are inflammatory conditions that affect the tissues around dental implants. They can be classified as peri-implant mucositis or peri-implantitis. Both are plaque-associated pathological conditions occurring in tissues around dental implants; however, in peri-implantitis, there is a progressive loss of supporting bone leading to the loss of the dental implant. Overdentures can easily accumulate plaque, stains, and calculus, especially on their attachment system. The cleaning of the abutment on locator-retained overdentures can be especially difficult. Food accumulation may occur in the shallow undercut of the locator abutment, thus making it harder to perform regular hygiene maintenance, as its cylindrical form will require more dexterity to brush all of its structure, especially closer to the gingival margin. This may be a factor of concern to the preservation of a healthy mucosa and to the hygiene maintenance of all of this attachment system’s components [13,14,15,16].

In order to control biofilms in the oral cavity, different oral hygiene products have been developed and marketed. Physical disruption and elimination of dental biofilms can be effectively accomplished with the use of mechanical devices and chemical agents as their applications (especially denture cleansers) to control denture plaque and bacteria levels, and several of these cleaning agents have been extensively evaluated. The efficacy of the different formulations has been reported in several systematic reviews [17,18,19,20,21,22,23,24,25].

The selection of these solutions must consider the microbial elimination effectiveness and the ability to preserve the oral rehabilitation constituent materials [17,21,22,23].

The aim of this study was to evaluate the effect of multiple denture cleaning solutions (Kukident^®^ (P&G Tech, Oxford Parkway, UK), Benfix^®^ (Laboratorios URGO S.L., Guipúzcoa, Spain), Corega^®^ (Stafford Miller, Waterford, Ireland), and Protefix^®^ (Neuhofer Weiche, Parchim, Germany)) and their influence on the deterioration and loss of retention on four different brands of overdenture attachments (OT Equator^®^ (Rhein83, Bolonha, Italy), Locator^®^ (Zest Anchors, Escondido, CA, USA), Kerator^®^ (KJ Meditech, Gwangiu, Republic of Korea), and Locator R-Tx^®^ (Zest Anchors, Escondido, CA, USA)) in a 12-month clinical use simulation.

## 2. Materials and Methods

### 2.1. Materials

All materials used in this study were selected based on their importance and usefulness in dentistry, as well as their stability under normal conditions of use and storage. All materials and chemicals were used in accordance with the manufacturers’ standards.

#### Materials Used in the Study

The overdenture attachment systems used in this study were OT Equator^®^ (Rhein83, Bolonha, Italy), Locator^®^ (Zest Anchors, Escondido, CA, USA), Kerator^®^ (KJ Meditech, Gwangiu, Republic of Korea), and Locator R-Tx^®^ (Zest Anchors, Escondido, CA, USA).

The cleaning solutions were selected due to their market recognition. These were Kukident^®^ (P&G Tech, Oxford Parkway, UK), Benfix^®^ (Laboratorios URGO S.L., Guipúzcoa, Spain), Corega^®^ (Stafford Miller, Ireland), and Protefix^®^ (Neuhofer Weiche, Parchim, Germany).

### 2.2. Methods

To test all of the selected products, a standard laboratory protocol was established and applied at the Laboratory of Investigation in Oral Rehabilitation and Prosthodontics, UNIPRO Oral Pathology and Rehabilitation Research Unit, University Institute of Health Sciences (IUCS), CESPU, Gandra, Portugal.

#### 2.2.1. Preparation of the Samples

**Figure 1:**
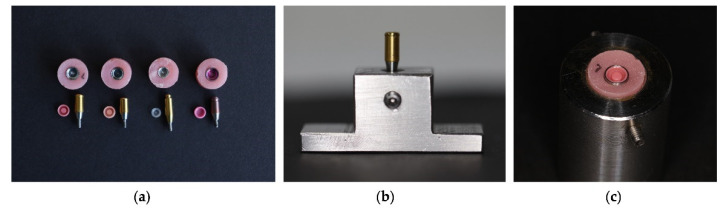
(**a**) Attachment system from each brand; (**b**) transfer table with the attachment abutment connected to the implant analog; and (**c**) upper block of the jig housing the denture caps of the overdenture attachment.

The samples consisted of 10 Teflon retentive caps from four different brands, and each cap was cleaned by each cleaning solution. In the study, abutments and metal housings from the respective brands were used (Figure 1a). Four cleaning solution brands were chosen for this test, and a control group was established using tap water. The retentive forces for each brand were selected based on the reference values from Locator^®^, as represented in pink in Table 1, and the retentive caps were also selected considering similar force values from other brands without angulation.

Therefore, a total of 200 samples of retentive caps and 5840 hygiene tablets were analyzed. Ten specimens of each brand of retentive caps were immersed in four different cleaning solutions to simulate 365 days of daily usage (Table 2).

#### 2.2.2. Preparation of the Acrylic Testing Block

An attachment abutment was connected to the implant analog at the center of the lower platform. Then, this attachment was manually tightened to the implant analog with 35 Ncm of torque using a screwdriver and ratchet torque controller from each brand, as shown in Figure 1b.

The upper block of the jig was used to assemble the denture caps of the overdenture attachment system and to test the nylon insert, which allows for replacement after each test. The metal housing (4 mm in depth) was indexed to the implant analog with a “direct” pick-up technique using auto-polymerizing poly-methyl methacrylate (Figure 1c).

#### 2.2.3. Protocol for Immersion in Cleaning Solutions

The different branded Teflon retentive caps (Table 1) were immersed in cleaning solutions for a period of time that simulated 365 days of daily oral hygiene, according to each manufacturer’s instructions. Then, the caps were subjected to retention tests (Table 3 and Table 4).

**Figure 2:**
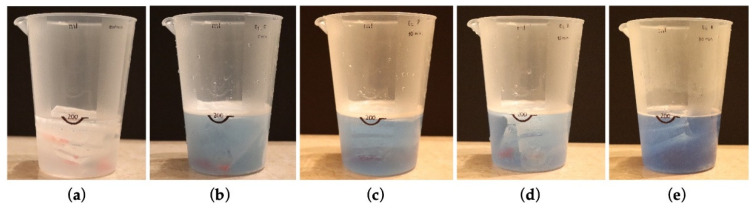
Attachments soaking in: (**a**) water; (**b**) Corega^®^; (**c**) Protefix^®^; (**d**) Benfix^®^; and (**e**) Kukident^®^.

The test and control groups subjected to immersion were carried out at room temperature (23 °C ± 2 °C). The attachments were placed in perforated plastic bags with a small marble used as a weight to ensure that the perforated bags would be immersed in the solutions for the entire soaking period. Each tablet was then dissolved in 200 mL of water at a temperature of 35 °C ± 2 °C and prepared according to the manufacturers’ directions (Figure 2, Table 5).

Following each immersion, the specimens were removed from the solution, rinsed in running water (15 s), and dried. Then, a new solution was prepared, and the procedure was repeated daily. Immersion procedures were repeated 365 times to simulate 365 days, according to the illustrative protocol.

Tap water was used as the control group (Table 6). This allowed for monitoring the influence of the cleaning solutions on the wear of the prostheses.

#### 2.2.4. Dynamic Fatigue Test

Once each group was submitted to a cycle of 365 daily immersion procedures, the samples were incorporated in the Instron^®^ (Norwood, MA, USA) testing machine with the titanium transfer table, to analyze the retention force over 1095 crosshead movements simulating 12 months of use. The Instron^®^ (Norwood, MA, USA) Electropuls E10000 LT testing machine is a dynamic fatigue testing machine with a 10 KN linear dynamic capacity, a 7 KN linear static capacity, a 60 mm linear stroke, and a 100 Nm torque capacity that allows for static, dynamic axial, and torsion tests in accordance with the ISO 7500-1 standard. It has an accredited calibration force of up to 5 meganewtons according to ISO 7500-1 and ASTM E4.

The maximum peak load-to-dislodgement was recorded automatically using the machine’s software. Assuming that overdenture users remove and insert their overdentures at least three times during the day, the study was carried out based on three full cycles per day (insertion-removal-insertion). All specimens were subjected to 1095 dynamic cycles equivalent to 365 days, thereby simulating 1 year of daily immersions. The analyzed datasets comprise 12 months of use, each corresponding to the arithmetic mean of 1095 consecutive insertion and removal cycles. The simulation was performed at a rate of 10 cycles per minute and at a constant speed of 50 mm/s, according to the estimated speed that patients remove their prostheses [26]. Each retentive cap insert was subjected to the same number of load cycles, controlled by the computer software, which was programmed to produce 1095 crosshead movements, with a sine waveform pattern, 1.4 mm vertical range, and 4 Hz frequency.

Prior to each test, the upper block that housed the nylon insert was displaced to the lower position until a contact was established, in order to ensure the accurate alignment to the attachment abutment on the lower block. Each retentive cap was fit onto the metal housing, then it was removed after each cycle, using an inserter/extractor tool from each brand.

All of the test results were recorded using WaveMatrix™2 test software version 2.0 (Instron^®^, Norwood, MA, USA), which facilitated the definition and execution of the tests and data acquisition. Next, all values and data were transferred to Microsoft Office Excel^®^, version 16.0 (Redmond, WA, USA), which was used to perform the statistical data analysis. The forces were recorded in Newton units (N).

### 2.3. Statistical Analysis

A sample of 200 retentive caps was determined based on power analysis for the expected number and nature of parameters to analyse differences in retention forces. This total sample will be distributed as it follows: (i) 10 retentive caps to immerse in Corega^®^ (Stafford Miller, Waterford, Ireland), (ii) 10 retentive caps to immerse in Protefix^®^ (Neuhofer Weiche, Parchim, Germany)), (iii) 10 retentive caps to immerse in Benfix^®^ (Laboratorios URGO S.L., Guipúzcoa, Spain), (iv) 10 retentive caps to immerse in Kukident^®^ (P&G Tech, Oxford Parkway, UK), and (v) 10 retentive caps to immerse in tap water.

For the descriptions of the continuous variables, the following descriptive statistics were used: count, mean, standard deviation, median, and interquartile range.

A two-way ANOVA was used to model the retention as a function of the cleaning solutions and attachment retentive caps. A Tukey HSD test was also performed to provide numerous pairwise comparisons between the means of the groups and categories. Moreover, a Levene Test and Shapiro–Wilk tests were used to assess the validation of the ANOVA assumptions. The statistical analysis was implemented using R version 4.2.2 software with the significance level set to *p* < 0.05.

## 3. Results

The descriptions of the continuous variables allow us to know the mean and standard deviation values: tap water (6.92 ± 2.72); Corega^®^ (Stafford Miller, Waterford, Ireland) (6.48 ± 2.95); Protefix^®^ (Neuhofer Weiche, Parchim, Germany) (6.81 ± 2.81); Benfix^®^ (Laboratorios URGO S.L., Guipúzcoa, Spain) (5.95 ± 2.73); Kukident^®^ (P&G Tech, Oxford Parkway, UK) (5.65 ± 2.39); OT Equator^®^ (Rhein83, Bolonha, Italy) (9.76 ± 1.40), Locator^®^ (Zest Anchors, Escondido, CA, USA) (7.59 ± 1.26), Kerator^®^ (KJ Meditech, Gwangiu, Republic of Korea) (4.50 ± 1.21), and Locator R-Tx^®^ (Zest Anchors, Escondido, CA, USA) (3.60 ± 0.93).

According to the two-way ANOVA results, the retentions values were significantly affected by the cleaning solutions and the attachment retentive caps (*p*-value < 0.05). Additionally, we may also deduce that the attachment retentive caps were the most important variable factor, since they presented a higher F value (Table 7).

The mean (±SD) retentive values for the attachments for each cleaning solution can be observed on Table 8.

The results of the Tukey HSD test showed that the differences between the attachment retentive cap brands were statistically significant, with an adjusted *p*-value of less than 0.05 for all pairwise comparisons (Table 9).

**Figure 3:**
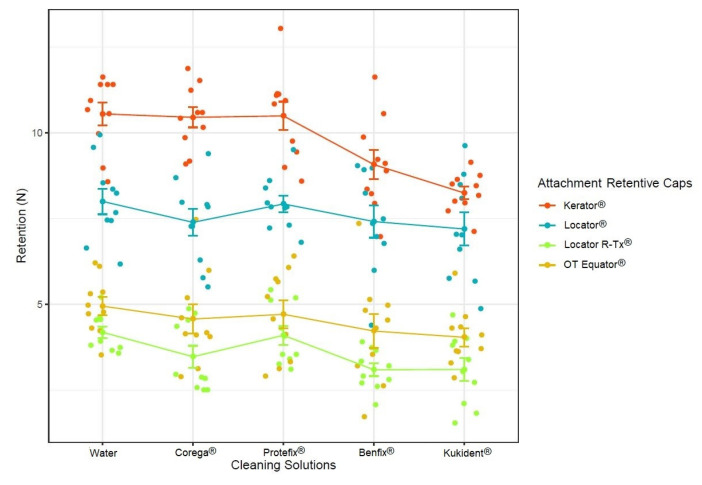
Retention of the attachments after they were soaked in different solutions.

Looking at the cleaning solutions group, significant statistical differences between attachment brands were only found between these specific brands: Kukident^®^–Corega^®^ (*p* < 0.05), Kukident^®^–Protefix^®^ (*p* < 0.05), water–Kukident^®^ (*p* < 0.05), Benfix^®^–Protefix^®^ (*p* < 0.05), and water–Benfix^®^ (*p* < 0.05) (Figure 3).

Using the Levene test, we found that the variations between the different groups were homogenous because no statistically significant results (*p*-value = 0.2684) were found. Additionally, no evidence of any normality violation was found (W = 0.99, *p* = 0.7728).

## 4. Discussion

Overdentures are removable dental prostheses that can be soft-tissue-supported implants. In other words, these prostheses can be used as supports for both implants and soft tissue, or for natural teeth or roots [27,28].

The clinical circumstances determine the selection of the attachment, since each has its own mechanical properties and load distribution characteristics. Moreover, prosthetic complications and maintenance also influence the attachment system selection. Therefore, this selection should be made following the proper identification of the individual’s intraoral structures, such as bone type and inter-arch space [5,27,29].

The quality and mechanical properties of the attachment system used on overdentures are some of the most important factors for improving patient satisfaction, retention, phonetics, and mastication. Therefore, knowing which factors influence the behavior and longevity of the overdenture components is crucial—attachment material, design, treatment surface, insertion and removal cycles, parafunctional habits, patient’s saliva pH, type of nutritional diet, types of drinks, and temperature variations [30,31,32,33].

Many studies show that cleaning solutions can lead to an increase in hardness and surface roughness following oral rehabilitation. This may be related to the possible loss of soluble components, such as polymers, acrylics, and metals, leaving empty spaces, corrosion, degradation, and discoloration [20,24,32,34].

Since it is fundamental to ensure a better durability of the components over the long term, many studies have been carried out to evaluate the impact of cleaning solutions on the retention of the overdenture attachments [35,36,37,38,39,40].

Commercially available chemical denture cleaners use various active agents, such as peroxides, hypochlorite, acids, and enzymes [17,18,19,20,32,41].

According to Ayyıldız et al.’s 2020 study, Corega^®^ (Stafford Miller, Waterford, Ireland), Protefix^®^ (Neuhofer Weiche, Parchim, Germany), and tap water all reduced the retention of Locator^®^ (Zest Anchors, Escondido, CA, USA) pink attachments by similar amounts and for all time intervals (1, 6, and 12 months). In addition, the results of that study also showed that the loss of retention values was higher in the sodium hypochlorite (NaOCl) solution group, followed by the group subjected to tap water. In contrast, in our study, the group of attachments immersed in water had the lowest loss of retention. This may be explained by the difference in the ion constitution of the water used in this study, classified as soft water (0-60 mL/CaCO_3_) [42]. Ayyıldız et al. suggest that the loss of retention caused by the tap water may be due to the metal ions, such as calcium and magnesium, and chlorine, as well as due to the pH values of the water. When the water has a higher ion concentration (hard water), it can induce deposit formation and inhibit the adequate fit of the attachment with the abutment that can result in permanent retentive property loss [36]. Similar to Ayyıldız et al.’s study, we were not able to find a statistically significant difference between Corega^®^ (Stafford Miller, Ireland), Protefix^®^ (Neuhofer Weiche, Parchim, Germany), and tap water, despite the retention reduction observed in all of them.

All of the studies that evaluated the influence of cleaning solutions on the retention of overdenture attachments and that included NaOCl as one of the cleaning solutions for evaluation concluded that this solution leads to the highest loss of attachment retention values. This compound was not included in our study due to the lack of professional advice regarding the use of this solution as a hygiene solution for dental rehabilitation. Additionally, NaOCl is associated with some changes in the morphology of the polyamide surface that leads to the creation of porosities and cracks and causes a loss of retention in the attachment’s materials [32,35,36,37,38,39,40].

According to Nguyen et al. 2010, the retention of Locator^®^ pink attachments was unaffected when soaked in Polident Regular^®^ (soaked for 3 m) and Polident Overnight^®^ (soaked for 8 h). This may suggest that the time of soaking does not have an influence on the retention of the attachment system [38]. In You et al.’s 2011 study, the attachments soaked in Efferdent^®^ for 15 m daily had a greater retention loss than the attachments soaked in Polident^®^ for 8 h daily, despite the lack of any statistically significant differences between the two groups [35]. However, in our study, statistically significant differences were found in the retention forces of the Benfix^®^ (Laboratorios URGO S.L., Guipúzcoa, Spain) and Kukident^®^ (P&G Tech, Oxford Parkway, UK) retentive caps compared to the control group. Those results are contradictory with Nguyen et al. 2010 and You et al.’s 2011 studies, which may suggest that the time of immersion in the cleaning solution could have an influence, since this was the main difference from the other solutions [35,38]. Despite this hypothesis, our results also show that the attachments subjected to Corega^®^ (Stafford Miller, Ireland) for 5 m were more affected than those subjected to Protefix^®^ (Neuhofer Weiche, Parchim, Germany) for 10 m, which is contradictory to the previous statement. However, this fact may be related to the effervescence time of the tablet, as the Corega^®^ (Stafford Miller, Ireland) tablet dissolves very quickly while the Protefix^®^ (Neuhofer Weiche, Parchim, Germany) tablet often takes more than 10 min to completely dissolve.

There are no previous studies in the field of dentistry that compare different brands of attachment systems with different cleaning solutions. The results obtained here show that there are statistically significant differences in the retention forces of the attachment retentive caps made by different manufacturers. However, in this study, the initial retentive forces are different between all of the groups, and although they are made of the same material, there are different compositions; therefore, each one has a different elasticity and consequent retention capability [32]. Consequently, these should not be the most relevant results, since the main objective was to observe the influence of the cleaning solutions on the retention and degradation of the different brands of overdenture attachments. In this way, it is possible to know which are the most recommended tablets on the market.

With the results of this study, it can be concluded that the denture cleaners that influence the retention forces of the retentive caps were statistically significant. However, comparing the control group with those subjected to cleaning solutions, significant statistical differences were found only between two groups (Benfix^®^ (Laboratorios URGO S.L., Guipúzcoa, Spain) and Kukident^®^ (P&G Tech, Oxford Parkway, UK)). Similar to other studies, our results showed statistically significant differences between Kukident^®^–Corega^®^, Kukident^®^–Protefix^®^, water–Kukident^®^, Benfix^®^–Protefix^®^, and water–Benfix^®^ in terms of their effects on the retention forces of the attachment retentive caps [35,36,37,38].

It is necessary to bear in mind that this in vitro study has several limitations. Patients can remove and insert their overdentures more frequently than three times a day and physical changes in the abutment and the attachments can occur during the testing procedure. Additionally, on a daily basis, intervals of overdenture maintenance are interrupted by intervals of usage, while in this study, the attachment caps were continuously immersed in solution for a simulated period of 12 months followed by simulated cycles of function.

This study simulated a 12-month period of daily oral hygiene and overdenture use; however, similar to Ayyıldız et al.’s 2020 study, further investigation with longer periods of time is necessary [35,36,37,38,39,40].

## 5. Conclusions

The present study concludes that the retention values were significantly affected by the cleaning solutions and the attachment retentive caps. Moreover, the results also determined that:

1. There were no significant statistical differences between Corega^®^ (Stafford Miller, Waterford, Ireland), Protefix^®^ (Neuhofer Weiche, Parchim, Germany), and tap water, despite the retention decreasing in all three solutions.
2. The only statistically different results found were between the Kukident^®^ (P&G Tech, Oxford Parkway, UK) and Benfix^®^ (Laboratorios URGO S.L., Guipúzcoa, Spain) cleaning solution groups, suggesting that the amount of time required for the cleaning solution to work could influence the attachment retentive cap’s degradation.
3. There were significant statistical differences between the different manufacturers in terms of the retention forces of the attachment retentive caps, despite the fact that the caps are made of the same material. There were different components that caused each one to have a different elasticity, resulting in retention differences, and explaining the variation between the initial retentive forces from all of the groups.
4. Further studies are necessary to analyze whether the percentage of different material elements used to make the attachment influence or accelerate the attachment retentive cap’s degradation.

Regarding the results, dentists should advise their patients with overdentures featuring this type of attachment system to use denture cleaners that require a shorter immersion time to ensure the longevity of all their attachment’s components.

## Figures and Tables

**Table 1:** Retentive caps chosen from each brand.

Brand	Color	Force
Locator^®^	Pink 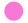	1360 g
OT Equator^®^	Clear 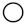	1300 g
Kerator^®^	Pink 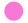	1088 g
Locator R-Tx^®^	Pink 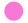	907 g

**Table 2:** Subdivision of the materials needed.

	Locator^®^	OT Equator^®^	Kerator^®^	Locator R-Tx^®^
Corega^®^ (1460 tablets)	10	10	10	10
Benfix^®^ (1460 tablets)	10	10	10	10
Protefix^®^ (1460 tablets)	10	10	10	10
Kukident^®^ (1460 tablets)	10	10	10	10
Control	10	10	10	10
Total	50	50	50	50

**Table 3:** Simulation of immersion periods in the cleaning solutions.

	Daily Hygiene (1 Day)	One Year (365 Days)
Corega^®^	5 min	1825 min
Protefix^®^	10 min	3650 min
Benfix^®^	15 min	5475 min
Kukident^®^	30 min	10,950 min

**Table 4:** Manufacturers’ specifications for immersion protocols.

Corega^®.^	Dissolve one Corega Cleanser^®^ tablet in warm (not hot) water to cover the denture.	For an antifungal action, leave it submerged for 5 min. You can also leave it overnight.	Rinse the denture with plenty of running water before putting it in your mouth.
Protefix^®^	Dissolve one Protefix Active Cleanser^®^ tablet in a glass of lukewarm water (100–200 mL, about 35 °C).	Clean and fresh in 3 min, disinfected in 10 min. Cleaning is also possible overnight.	Rinse the dental prosthesis well with running water before putting it in the mouth.
Benfix^®^	Introduce a single cleaning tablet in a glass of warm water.	Let the product act for a minimum of 15 min. For deep cleaning, you can leave your denture in the cup overnight.	Rinse with plenty of water to eliminate possible product residue.
Kukident^®^	Put the tablet in enough warm water to cover the denture.	Place the denture in the solution and let it sit for 30 mor overnight.	Remove the dentures and rinse in plenty of running water.

**Table 5:** Experimental design and soaking periods.

	Locator^®^		Kerator^®^		OT Equator^®^		Locator R-Tx^®^	
	Time	Solution	Time	Solution	Time	Solution	Time	Solution
Control (water)	-	-	-	-	-	-	-	-
Experiment 1	5 min	Corega	5 min	Corega	5 min	Corega	5 min	Corega
Experiment 2	10 min	Protefix	10 min	Protefix	10 min	Protefix	10 min	Protefix
Experiment 3	15 min	Benfix	15 min	Benfix	15 min	Benfix	15 min	Benfix
Experiment 4	30 min	Kukident	30 min	Kukident	30 min	Kukident	30 min	Kukident

**Table 6:** Tap water composition.

Tap Water Composition	Data
pH (Sørensen’s scale)	6.9–8.7
Hardness (mg/L CaCO_3_)	38
Calcium (mg/L Ca)	13–16
Magnesium (mg/L Mg)	0.52–0.75
Chlorine (mg/L Cl_2_)	0.01–1.02

**Table 7:** Two-way ANOVA summary. Retention as the function of the cleaning solutions and attachment retention caps.

	Df	Sum Sq	Mean Sq	F Value	Pr (>F)
Cleaning solutions	4	48.1	12.0	9.616	4.15 × 10^−7^ ***
Attachment retentive caps	3	1208.6	402.9	322.066	<2 × 10^−16^ ***
Residuals	192	240.2	1.3	10	

Significance codes: 0 ‘***’ 0.001.

**Table 8:** Description of the cleaning solutions per attachment retentive caps—mean and standard deviation of each cleaning solution/attachment retentive caps.

**Cleaning Solution** **Attachment System**	**Water (Control)**	
	**Mean**	**SD**
Locator^®^	8.00 N	1.18 N
Kerator^®^	10.6 N	1.07 N
OT Equator^®^	4.95 N	0.834 N
Locator R-Tx^®^	4.19 N	0.534 N
**Cleaning Solution** **Attachment System**	**Corega^®^**	
	**Mean**	**SD**
Locator^®^	7.39 N	1.24 N
Kerator^®^	10.5 N	0.926 N
OT Equator^®^	4.58 N	1.35 N
Locator R-Tx^®^	3.48 N	1.01 N
**Cleaning Solution** **Attachment System**	**Protefix^®^**	
	**Mean**	**SD**
Locator^®^	7.93 N	0.769 N
Kerator^®^	10.5 N	1.31 N
OT Equator^®^	4.71 N	1.29 N
Locator R-Tx^®^	4.10 N	0.871 N
**Cleaning Solution** **Attachment System**	**Benfix^®^**	
	**Mean**	**SD**
Locator^®^	7.42 N	1.49 N
Kerator^®^	9.07 N	1.34 N
OT Equator^®^	4.23 N	1.56 N
Locator R-Tx^®^	3.10 N	0.580 N
**Cleaning Solution** **Attachment System**	**Kukident^®^**	
	**Mean**	**SD**
Locator^®^	7.20 N	1.53 N
Kerator^®^	8.25 N	0.578 N
OT Equator^®^	4.05 N	0.843 N
Locator R-Tx^®^	3.11 N	1.04 N

**Table 9:** Family-wise confidence interval for the Tukey 95% multiple comparison and the *p*-value after the multiple comparisons adjustment. Mean of the maximum force (F max) required to dislodge from the attachment abutment.

	Fmax (Mean ± SD)	Multiple Comparison Results
1. Kerator^®^	9.76 ± 1.40 N	1 vs. 2 (<0.001) 1 vs. 3 (<0.001) 1 vs. 4 (<0.001)
2. Locator^®^	7.59 ± 1.26 N	2 vs. 3 (<0.001) 2 vs. 4 (<0.001)
3. OT Equator^®^	4.50 ± 1.21 N	3 vs. 4 (<0.001)
4. Locator R-Tx^®^	3.60 ± 0.93 N	

## Data Availability

Data that support this study’s findings are available from the corresponding author upon request.
